# Bromide: the good, the bad, and the ugly of the oldest antiseizure medication

**DOI:** 10.3389/fvets.2024.1433191

**Published:** 2024-06-26

**Authors:** Diogo Gouveia, Paul Mandigers, Giunio Bruto Cherubini

**Affiliations:** ^1^Dick White Referrals – Linnaeus, Cambridgeshire, United Kingdom; ^2^Department of Clinical Sciences, Faculty of Veterinary Medicine, Utrecht University, Utrecht, Netherlands; ^3^Department of Veterinary Sciences, Veterinary Teaching Hospital “Mario Modenato”, University of Pisa, Pisa, Italy

**Keywords:** epilepsy, seizure, canine, adverse effects, salt, refractory epilepsy, ASM

## Abstract

Bromide is the first effective antiseizure medication used in human medicine since the XIX century. Initially met with skepticism, bromide quickly gained enthusiasm within the medical field until being largely replaced by newer antiseizure medications with significantly fewer adverse effects in people. In veterinary medicine, bromide continues to be used in the management of epileptic patients for over 30 years, yet adverse effects can impact owners and patients alike. We sought to provide the general practitioner and veterinary neurologist with insightful information on both the positive and negative attributes of bromide, explore factors that may influence its desirability as an antiseizure medication in specific veterinary cases and elucidate its current role in modern epilepsy treatment for veterinary patients. It’s also our endeavor to discuss the current use as an alternative or add-on with other known antiseizure medications and potential future studies that might enhance our understanding and use of this medication.

## 1 Introduction

Epilepsy is the term used to describe a brain disease that leads to a persistent predisposition for the generation of epileptic seizures, with this term being applied to patients that experience at least two unprovoked epileptic seizures separated from more than 24 h (1).

A systematic review and metanalysis by Fiest et al. (2) identified a point prevalence of active epilepsy of 6.38 per 1,000 people.

In canine patients, epilepsy was found to be the most prevalent chronic neurological disorder (3, 4). Although the exact point prevalence is not known, this was estimated to be between 0.6 and 0.75% in the general dog population (1). Epilepsy can also occur in cats. O’Neil et al. (5) reported epilepsy with a one-year period prevalence of 0.04% for patients presenting to primary veterinary care practices in the United Kingdom.

Idiopathic epilepsy (IE), which can be further subclassified into genetic epilepsy, suspected genetic epilepsy or IE of unknown cause, is the most common cause responsible for seizures in dogs (1, 4). Seizures can also be a consequence of intracranial disease (structural epilepsy), metabolic disorders or intoxication (reactive seizures) (6).

Treatment of patients suffering from epilepsy is recommended according to the patient’s seizure frequency, severity of ictal or post ictal phase, owner’s beliefs, and lifestyle (7). Adequate seizure control can improve the quality of life for both canine patients and their owners (8). In addition to this, seizure frequency might increase overtime in untreated patients suffering from idiopathic epilepsy, emphasizing another possible advantage of initiating treatment in a timely fashion (Table 1) (7, 10).

**Table 1:** International veterinary epilepsy task force (IVETF) current indications to recommend maintenance ASM treatment (9).

Interictal period equal or inferior to 6 months
Status epilepticus or cluster seizures
Severe postictal signs (e.g., aggression) or lasting longer than 24 h
Increasing seizure frequency and/or duration and/or severity over 3 interictal periods

The use of antiseizure medications (ASMs) is the current mainstay for the treatment of epilepsy.

Phenobarbital is the oldest and most commonly used ASM in veterinary patients (11). It is well tolerated, has a widespread availability and low cost, making it the primary treatment choice in canine epilepsy (10, 11). In addition to phenobarbital, imepitoin and potassium bromide are the only approved ASM for the treatment of canine epilepsy in Europe (11). In the United States potassium bromide (KBroVet-CA1) and phenobarbital (Fidoquel-CA1) are currently the only two medications that received conditional approval by the Food and Drug Administration (FDA) in January 2021 and September 2023, respectively (9, 12). The use of primidone, levetiracetam, zonisamide, felbamate, topiramate, gabapentin, pregabalin is also described, although the levels of evidence supporting their efficacy vary considerably (13).

Bromide is the name given to a negatively charged bromine ion (14). It is usually administered associated to potassium, in the form of potassium bromide (KBr) and can be used in monotherapy or as an add-on ASM (15).

Bromide has now been successfully used as an ASM in the treatment of canine epilepsy for more than three decades (14, 16).

The aim of this paper is to give a historical perspective of the use of bromide in the treatment of epilepsy in veterinary and human medicine, review its mechanisms of action, pharmacokinetics, currently reported efficacy, known adverse effects and tolerability, possible drug interactions, current indications for serum level monitoring and have an outlook on how it currently compares with other frequently used ASMs. The present research gaps and potential future developments in the use of this medication are also reviewed and discussed.

The information presented in this review aims to enhance the understanding of primary practitioners and veterinary neurologists regarding the current insights into the use of bromide in epileptic patients. It seeks to raise awareness about both the positive and negative aspects of this substance, empowering clinicians to make more informed decisions when utilizing this well-established ASM.

## 2 Historical perspective

In human medicine, bromide was recognized as an effective antiepileptic medication in 1861, after being previously used in 1857 by Sir Charles Locock, an obstetrician for Queen Victoria, to treat young woman with “hysterical” epilepsy and “catamenial seizures,” connected with the menstrual period (17–20).

Bromide was previously better known for its efficacy in improving clinical signs associated with nymphomania or priapism, and its newly discovered antiseizure effects only got more attention at a later stage, in the mid-1860s (21).

Despite the lack of any randomized studies at that time, the available clinical data is considered enough to name bromide as the first effective ASM, marking the beginning of the modern treatment of human epilepsy (19).

After the initial use of potassium bromide, other different formulations were also tried, including its association to sodium or ammonium. Neither of the attempted alternatives revealed increased efficacy in the control of seizures or decreased adverse effects, which became one of the main drawbacks of bromide therapy (21). Reckless use of this medication with excessively high doses and lack of adequate follow-up were also one of the suspected reasons behind the development of unacceptable mental dullness and apathy in people (21). Adverse effects affecting the patient’s skin also became well recognized, with the development of cutaneous eruptions (bromoderma) that added significant morbidity to human patients treated with this medication (22).

Bromide continued to be the only effective ASM used in human medicine until 1912, when the antiseizure properties of phenobarbital were discovered (18). When compared to bromide, the decreased sedative effects associated with phenobarbital and newer molecules made them to gradually replace and be preferably used (18, 23).

Despite having a currently more limited role in human epilepsy, bromide can still be considered and have an important role in the treatment of refractory pediatric epilepsy (24, 25).

Schwartz-Porsche and Jürgens (26) described the use of potassium bromide as an effective add-on therapy (in addition to phenobarbital and/or primidone) to treat canine patients with refractory epilepsy for the first time in veterinary medicine in 1991. In this study, bromide led to an improvement in seizure control in 11 of the 22 dogs that composed the cohort, 4 become seizure free and 7 had a reduction of seizure frequency of at least 50% (26).

Podell and Fenner (23) and later Trepanier et al. (27) provided further evidence for the usefulness of bromide in the treatment of canine epilepsy, with studies revealing an overall efficacy of 83 and 72% in reducing seizure frequency and exponentially increasing the interest of this substance in veterinary epilepsy.

Following its initial use as an add-on therapy in association with phenobarbital, reports on the use of bromide as an effective alternative first-line antiepileptic also became available (16, 28).

## 3 Mechanism of action and pharmacokinetics

Bromide’s mechanism of action seems related to its preferential movement across neuronal chloride channels. This facilitates its intracellular accumulation in the neurons and increased gamma-aminobutyric acid (GABA) inhibition due to hyperpolarization of the membrane (29–32). Medications like barbiturates (e.g., phenobarbital), which boost chloride conductance through GABA-ergic activity, may work together with bromide to elevate the seizure threshold (29).

Although an active transport system in the choroid plexus can remove accumulation of bromide from the CSF and CNS, this system can be overwhelmed if administration of bromide is high enough (33).

GABA release, binding, transport or metabolism, however, does not appear to be affected by acute or chronic exposure to bromide (34).

After oral administration, bromide is easily absorbed in the gastrointestinal tract and has an estimated mean bioavailability of 46% (35), and maximal concentration in the cerebrospinal fluid occurs in about 2 h (36). In contrast with other antiseizure medications, bromide does not go through hepatic biotransformation and has no hepatotoxic effects (29). Canine thyroid function also does not seem to become affected by the chronic use of bromide (37, 38).

The unchanged halide of bromide is eliminated in the urine after being filtered from the bloodstream at the level of the glomerulus (39). After glomerular filtration, bromide suffers extensive tubular reabsorption leading to its long elimination half-life (39). Elimination half-life is variable and reported between 15 (40) and 46 days in dogs (35), approximately 11 days in cats (41) and 12 days in humans (42).

As with any medication, a steady-state serum concentration is reached after about four to five eliminations half-lives of regular administration (43). For this reason, in canine patients, bromide steady-state concentrations are expected to take 2–3 months to achieve (44). Despite this, therapeutic serum concentrations can still be achieved before reaching steady-state concentrations, what can justify the administration of initial loading doses in selected patients (35).

Chloride and bromide compete for tubular reabsorption, with bromide being naturally more easily reabsorbed and chloride more easily excreted (39, 45). An increase in chloride intake can enhance bromide renal elimination (35, 39, 46). Changes in chloride intake can occur with changes in dietary salt (sodium chloride) content.

Mercurial diuretics can increase bromide elimination, suggesting that bromide reabsorption might occur via the chloride channels in the thick ascending limb of Henle (47, 48). Loop diuretics like furosemide might also increase bromide elimination by blocking chloride and bromide reabsorption (49, 50). Osmotic diuretics have no impact on bromide elimination (39).

## 4 Dosage and administration routes

Bromide is typically administered orally in the form of the salt potassium bromide (KBr) (51). Sodium bromide (NaBr) constitutes an alternative, less common formulation that can also be used in veterinary patients. Currently, the use of NaBr is limited by the absence of commercially available formulations. Dosage must be adapted accordingly to the salt that is being used. The dose for NaBr is approximately 15% less of that of KBr, since potassium has a higher molecular weight than sodium, making 1 g of NaBr to contain more bromide than 1 g of KBr (28, 44, 51). Sodium bromide can be particularly useful in dogs with disease where administration of potassium might be undesirable (e.g., hypoadrenocorticism) but is contraindicated in case of hypertension, congestive heart failure or hepatic disease (28).

In canine patients, potassium bromide dosage as add-on therapy (e.g., in association with phenobarbital) is 20–40 mg/kg/day (11, 26, 28, 44, 51). Doses between 30 and 40 mg/kg/day can be adequate when using bromide in monotherapy (11, 51, 52). Although Trepanier (28) reported doses between 50 and 80 mg/kg/day to be possibly needed in some patients treated with bromide alone, the authors believe these doses are most likely excessively high and not indicated in most patients. Due to bromide’s long elimination half-life, dosing can be performed only once daily, what might help increasing owner’s compliance (44).

A dosage of 30 mg/kg/day was suggested for use in cats (41), but currently there is a weak level of evidence regarding the efficacy and safety of this medication in feline patients (53). The limited number of studies assessing bromide use in feline patients is likely related to the frequent adverse effects associated with its use in this species (as discussed in another section of this manuscript).

In horses, Raidal and Edwards (54) described the use of a loading dose of 120 mg/kg/day during a 5-day period and maintenance doses of 90–100 mg/kg of potassium bromide administered once daily. This protocol appears to lead to serum bromide concentrations that are associated with clinical efficacy for seizure control in other species.

Given bromide’s extended elimination half-life, a loading dose can be administered to reach the desired therapeutic serum concentrations levels faster in selected patients (14, 44, 51). Different loading dose protocols were previously described (14, 51, 55).

A loading dose of 125 mg/kg, divided into two daily administrations over 5 days, has been previously suggested. This approach results in a total loading dose of 625 mg/kg by the end of the 5-day period (51). After the end of the loading period, dogs should continue receiving the normal maintenance bromide dose.

Alternatively, in an emergency (e.g., patients in status epilepticus) the loading dose of 600 mg/kg can be administered quicker, over a 24-h period, in multiple 100 mg/kg dosages given 4 h apart (55). Given that loading doses may carry a higher risk of gastric irritation and vomiting, it is advisable to hospitalize patients during the loading dose period to enable better monitoring and control of these potential adverse effects. The previously described protocol can also be given rectally, in patients that are unable to take oral medication (56).

A study by Gindiciosi et al. (14) described a loading protocol that consisted of the oral administration 600 mg/kg of KBr split into multiple doses and given over a 48-h period in association with a maintenance dose of 30 mg/kg/day. This protocol was effective in achieving bromide therapeutic concentrations in most patients. Only 5% of the patients in this study vomited during the loading period, without this being impeditive to finish the loading protocol (14).

Longer loading periods with more fractioned dosages might reduce the risk of gastrointestinal signs (44). Patients should be regularly assessed during loading dose protocols for monitoring of side effects that might occur, allowing adjustment or discontinuation of the protocol if necessary.

A “mini” loading dose of 225–250 mg/kg was also suggested to provide rapid adjustments on bromide serum concentrations in patients where this might be required (44).

Intravenous loading can be performed with a NaBr solution and a protocol using a continuous rate infusion (CRI) during a 24-h period to administer a total of 900 mg/kg dose was previously suggested (51). Despite this, clinical studies evaluating the safety and efficacy of intravenous bromide administration in veterinary patients are still lacking.

Intravenous administration of KBr cannot be recommended due to the possible cardiotoxic effects associated with the rapid IV infusion of potassium (57).

## 5 Efficacy and comparative analysis with other antiseizure medications

Bromide was initially introduced in veterinary medicine as an “add-on” medication for the treatment of epileptic dogs refractory to treatment with phenobarbital and/or primidone, or for cases in which phenobarbital dosage needed reduction due to liver disease (23, 26, 58–60).

Improvement in seizure control (>50% reduction of seizure frequency) was recorded in 50–83% of the dogs where this medication was added to the antiseizure treatment, providing evidence of its usefulness for this purpose (23, 26).

Reductions in phenobarbital dosage were possible in 35% (23) and 70% (58) of dogs, after the addition of bromide. In a study by Trepanier et al. (27) addition of bromide made possible to discontinue barbiturate treatment (phenobarbital or primidone) in 19% of the dogs.

Royaux et al. (61) described possible benefits of the addition of bromide to epileptic dogs receiving imepitoin. A response rate (decrease of at least 50% in monthly seizure frequency) of 69% (9/13) was reported with the addition of bromide to the treatment of imepitoin-refractory epileptic dogs (61). In 23% (3/13) of the patients, seizure eradication was possible to achieve (61).

The use of bromide in monotherapy, as a first-line antiseizure treatment in alternative to phenobarbital, was also previously described (28). Currently there is only one study evaluating bromide’s efficacy in monotherapy for the treatment of epilepsy in dogs and comparing it with phenobarbital (16). First line treatment with potassium bromide was considered to be an acceptable choice, leading to the eradication of seizures in 52% of the dogs (16). Despite this, when compared to bromide, phenobarbital revealed a higher efficacy, leading to the eradication of seizures in 85% of dogs. If the patients continued to experience seizures, these were more likely to decrease in duration for dogs treated with phenobarbital compared to those treated with bromide. In addition to this, and although not statistically significant (*p* = 0.059), the percentage of dogs in which seizures were successfully controlled (>50% reduction in seizure frequency, decreased severity and without unacceptable adverse effects) was also higher for those treated with phenobarbital (15/20; 75%) than for those treated with bromide (15/23; 65%). Improvement of initially witnessed side effects was reported with both medications, but phenobarbital still appeared to be better tolerated, with 20% of the patients receiving bromide continuing to experience vomiting by the end of the study (16).

According to Podell et al. (11), bromide receives a moderate recommendation for its’ use in monotherapy and this medication is “most likely” expected to provide an effective treatment.

Compared to canine patients, the efficacy of bromide seems to be less in cats (41). Boothe et al. (41) reported that the use of bromide as monotherapy (4) or in association with phenobarbital (3) lead to the eradication of seizures in 7 of 15 treated cats. However, cats may develop an eosinophilic bronchitis which makes bromide use in cats not advisable, what also limits the current available studies in this species (62).

Studies assessing the efficacy of bromide as an antiseizure medication in horses are currently lacking.

## 6 Adverse effects and tolerability

Neurological and behavioral signs were the most reported adverse effects among different veterinary species (63).

Sedation, ataxia and/or paresis can occur in patients treated with bromide, particularly with increased serum concentrations (14, 23). Controversially, irritability and restlessness were also reported with potassium bromide treatment in dogs (64).

Non-neurological adverse effects include mainly polyuria, polydipsia and polyphagia, all frequently seen in patients receiving phenobarbital (14, 23).

### 6.1 Polyphagia

Polyphagia might be related to an increased caloric need or behavioral effect associated with this medication (63). Podell and Fenner (23) noted polyphagia in 7/23 dogs treated with a combination of phenobarbital and bromide once the last one was added to the antiseizure plan. In these patients it was not possible to conclude if polyphagia was due to bromide or the combination of the two medications (23). Polyphagia was also reported by about 20% of the owners of dogs treated with bromide, with this number increasing to around 80% if this medication was combined with phenobarbital (64).

Although weight variations in patients treated with bromide are currently not clear, eating habits and body weight should be closely monitored (63). Polyphagia can be severe to lead to garbage and foreign body ingestion, leading to secondary complications (44, 63).

### 6.2 Polyuria and polydipsia

Evidence regarding the frequency of polyuria and polydipsia (Pu/Pd) in dogs treated with bromide is controversial. Podell and Fenner (23) reported Pu/Pd in 13 out of 23 dogs receiving potassium bromide in combination with phenobarbital (23). On the other hand, in a study by Chang et al. (64) although Pu/Pd was not noted by the owners of dogs receiving bromide in monotherapy, these adverse effects were more frequently reported in patients receiving multitherapy with phenobarbital and bromide, rather than phenobarbital alone, suggesting that bromide can have a potential for these adverse effects (64). On the other hand, Pu/Pd is a commonly reported adverse effect in the clinical experience of one of the authors (GBC).

### 6.3 Gastrointestinal signs

Vomiting and diarrhea (including bloody feces) were also described in dogs treated with potassium bromide or sodium bromide but are usually not severe and seldomly indicate discontinuation of treatment (63). Based on anecdotal reports regarding the gastric irritant effect of bromide salts (28), administration in divided smaller doses or in association with food might help preventing vomiting (44, 63).

Bromide was previously associated with an increased risk for the development of pancreatitis (23, 26, 65). Dogs receiving treatment with bromide seem to have a higher risk of having elevated serum canine pancreatic lipase (66) and Gaskill and Cribb (65) reported an increase incidence of pancreatitis from 0.3% to at least 10%, after adding potassium bromide to the treatment of epileptic dogs treated with phenobarbital.

Phenobarbital alone or in combination with bromide can also lead to the development of hypertriglyceridemia in dogs, a known risk factor for pancreatitis (67). Despite this, the impact of bromide in contributing to hypertriglyceridemia is currently unknown and evidence to support a clear causal relationship between bromide treatment and pancreatitis also seems to still be lacking.

Megaoesophagus has been anecdotally reported (68), although whether there is a causal relation with bromide treatment remains unclear.

### 6.4 Dermatologic signs

Dermatologic adverse effects are only rarely reported in canine patients and do not appear to be a significant problem in patients receiving bromide therapy (63). Cutaneous lesions included nonsuppurative white macules with scales and pustular dermatitis (63). Panniculitis was reported in two dogs and resolved after discontinuation of bromide treatment (69).

Pruritus not associated with skin lesions was also previously reported (44, 64).

### 6.5 Bromide toxicosis (bromism)

Bromide toxicosis (bromism) appears to be dose-dependent and linked to high serum bromide concentrations (63, 68).

Rossmeisl and Inzana (68) found a mean bromide serum concentration of 3.7 mg/dL in dogs with clinical signs of bromism, compared to 1.7 mg/dL in control dogs.

Despite this, bromide tolerance seems to vary among individuals and, as a result, cases of toxicosis were also reported with low serum concentrations (40). For this reason, clinical signs should always be used in association with serum bromide concentrations to appropriately judge the tolerability of bromide treatment in a specific patient (63).

Serum bromide concentrations should be regularly assessed to identify possible changes in concentration trends and allow intervention before the development of signs of bromism or breakthrough seizures (68).

Throughout different species, severe bromide toxicity results in manifestations such as depression, alterations in behavior, ataxia, hind limbs paresis, mydriasis, stupor, and coma (63).

In cases where bromide is given in association with phenobarbital, a decrease of 10–30% of the dosage of phenobarbital can reduce the severity of neurological adverse effects in a few days.

Bromide dose reduction, intravenous fluid therapy and induction of diuresis with saline solution (0.9% NaCl) can lead to a quicker improvement in a matter of hours (63, 68).

Breakthrough seizures might occur during the treatment of bromism, which might increase the patient’s hospitalization time. Care should be taken while administering intravenous fluid therapy and this should be performed in association with serial monitoring of serum bromide concentrations (68).

### 6.6 Other

Experimental studies in rats revealed that bromide can disturb the thyroid, testes and adrenal’s function (70, 71). Despite this, in the studies by Kantrowitz et al. (37) and Paull et al. (38) bromide revealed no influence in canine thyroid function or morphology.

Bromide should not be used in pregnant or lactating animals since its safety is yet to be assessed in these patients.

### 6.7 Non-canine patients

In feline patients, cough, is the most frequent adverse effect (53) this is associated with the development of an eosinophilic bronchitis that can be life-threatening (62).

In a retrospective study, Boothe et al. (41) reported adverse effects in 8 out of 17 feline patients receiving bromide. Cough was particularly common (6/17) and lead to the death of one patient, with an overall mortality rate of 5/17. In another study cough was described in 11/26 cats treated with bromide (72). Two of these patients died as a result for severe respiratory signs (72). Onset of cough was reported between 2 weeks to 23 months after starting treatment (41), but was seen to developed as late as 8 years after initiation of treatment (62).

Resolution of cough occurs only after discontinuation of treatment, supporting a relationship with bromide treatment (41, 62). However, time for resolution was reported to be very variable (1–16 months) (41). Corticosteroid treatment might be required to cease respiratory signs in some patients (41, 62) and some might require long-term corticosteroid medication even after cessation of bromide treatment, suggesting that bromide might lead to severe and irreversible chronic inflammatory lower airway disease in some feline patients (62). Nodular pulmonary lesions and endogenous lipid pneumonia with secondary pneumothorax were also previously described (62).

Respiratory adverse effects were rarely reported in clinical studies in canine patients (51, 63).

Dermatitis (bromoderma; idiosyncratic or dose-independent) and vomiting, weight gain and polydipsia (dose-dependent) were the most uncommon adverse effects reported in cats (53).

In horses, bromide has also a sedative and calming effect that can lead to its misuse in competition animals (73). In cattle bromide seems to help decreasing aggressive behaviors (74).

## 7 Serum level monitoring

Bromide serum concentration monitoring is indicated in patients receiving treatment with this medication. Adequate monitoring allows individualization and optimization of treatment due to the narrow therapeutic ranges and known pharmacokinetic variability between individuals (52).

Serum level monitoring also allows the clinician to determine if medication failure is related to metabolic tolerance (in patients requiring dose adjustment) or functional tolerance (in patients requiring a change of medications). It helps monitoring owner’s compliance and prevent possible toxic effects (75).

Bromide serum concentrations should be measured once a steady-state concentration is reached between 6 and 12 weeks after the beginning of treatment with a maintenance dosage (11, 51, 52). In patients showing good seizure control, bromide serum concentrations should be assessed on an annual basis. This assessment should be anticipated if seizure frequency increases with more than 3 seizures before the next scheduled assessment or if toxicity is suspected (11).

The end result of a loading dose can be assessed 1 week after the administration of a loading dose protocol. Subsequently, another assessment should be performed one-month post-loading to determine the appropriate ongoing maintenance dose (51, 52). The patient’s maintenance dose should be increased if there is a decrease of more than 10% in bromide serum concentration between these two measurements (76).

Due to bromide’s long elimination half-life, timing of blood sample collection after oral administration is not critical, although samples collected more than 2 h after dosing should help avoiding peak effect variability (35).

The current recommended therapeutic bromide serum concentrations are based in only a few previous studies on the use of this medication (23, 26, 27).

If used as an add-on treatment, in association with phenobarbital or primidone, bromide serum concentrations between 700 and 2,000 mg/L (26) and 880–2,470 mg/kg (23) proved to be effective in improving seizure control.

Similarly, Trepanier et al. (27) found serum concentrations between 810 and 2,400 mg/L to be adequate when bromide was used in association with phenobarbital, and 880–3,000 mg/L when bromide was used in monotherapy.

It is important to mention that the suggested bromide therapeutic serum concentrations should not be seen as an absolute truth. Prospective dose titrating studies are still currently lacking, and it is possible for adequate seizure control to occur with concentrations below those of the expected therapeutic range. Equally (as previously discussed), concentrations above this range do not necessary result in severe side effects or bromism in all patients (16).

To date, determining an accurate serum bromide concentration remains challenging, typically requiring samples to be sent to an external laboratory, which can potentially delay therapeutic decisions (77).

Most analytical methods that are currently used to assess chloride concentrations measure the total halide ion concentration, what includes chloride and bromide. This leads to spurious hyperchloremia (or pseudohyperchloremia) to be frequently recorded when assessing the serum, whole blood or plasma of patients receiving bromide treatment (77, 78).

A study by Woody et al. (79) showed that, in human patients, the degree of pseudohyperchloremia could be used as an indirect method to assess bromide serum concentration in a quicker manner (79). Inspired by these results, Rossmeisl et al. (77) studied the existence of a possible similar relationship between the magnitude of pseudohyperchloremia and bromide serum concentration in bromide treated epileptic dogs. Rossmeisl et al. (77) concluded that in dogs this relationship was unsatisfactory for routine use in practice and bromide dose adjustments should be based on the direct assessment of bromide serum concentration. Despite this, since chloride can easily be measured in-house in most practices, the variation and degree of hyperchloremia can still be used as a general guide to adjust bromide dosage in emergency situations (77).

Bromide concentration can be determined by a spectrophotometric, gold chloride method (80). After precipitation of the proteins in the sample, tri-gold chloride is added to the serum leading to a color change that is related to the level of bromide present in the blood. The resultant color change is then read spectrophotometrically (81). This method carries a risk of falsely record elevated bromide levels due to the presence of other iodides (78). Additionally, this method is laborious and nowadays most laboratories prefer the use of mass spectrometry.

Due to the impact of hemolysis in spectrophotometrical techniques (82), care to avoid erythrocyte damage should be taken when collecting blood samples for bromide measurement.

A study by Mandigers (83) assessed 51 dogs receiving bromide treatment in two different laboratories and revealed a difference between −1111 mg/L to 3,910 mg/L, with a mean difference of −128 ± 728 mg/L between the two laboratories. These results concerningly show that bromide measurements can differ very significantly between different laboratories, even when the same laboratory method is used, providing further evidence on the difficulty in obtaining reliable bromide measurement results.

Mass spectrometry is used to identify and quantify different types of molecules based on their mass-to-charge ratios. In simple terms, mass spectrometers measure the mass of molecules that were previously converted into ions, allowing their precise quantification (84). Once limited to research and specialized clinical laboratories, mass spectrometry is now more readily available and routinely used (85).

## 8 Drug interactions

Bromide has no known interactions with other common antiseizure medications (81).

Muñana et al. (86) assessed the possible influence of a combination of phenobarbital and/or bromide in the clearance of levetiracetam of epileptic dogs receiving multidrug treatment. The combination of bromide with levetiracetam did not result in any significant recorded pharmacokinetic interaction. This contrasted with the combination of phenobarbital (alone or in association with bromide) with levetiracetam, which led to an increased clearance and lower levetiracetam plasma concentrations (86).

The absence of known interactions between bromide and other antiseizure medications may be attributed to its lack of metabolism within the patient’s body (81).

Bromide exhibits its most significant and well-known interaction with chloride, which in turn is influenced by the patient’s diet. Diets with higher chloride content may result in faster elimination of bromide (87).

Trepanier and Babish (87) suggested that the predicted mean daily doses of bromide needed to maintain an adequate serum concentration were significantly higher for patients with higher chloride content in their diet (87). For this reason, there is a general recommendation for the ingestion of chloride to be maintained constant in human and veterinary patients receiving treatment with bromide (87, 88). Dogs with adequate seizure control can experience recurrence of seizures after a sudden increase in chloride intake through a diet change and consequent drop in serum bromide concentrations (89).

A study by Lichtenauer et al. (90) evaluated a possible relationship between the proximity of a dog’s residential area to the coast and the dose of potassium bromide required to maintain adequate serum bromide concentrations (90). It is thought that dogs living close to the sea can be exposed to air with higher concentrations of salt in the form of aerosols (91, 92). Despite the lack of statistically significant results, this study identified a trend where dogs living by the sea appeared to require higher doses of potassium bromide, suggesting that the impact of chloride in dogs treated with bromide might go beyond nutrition (90).

In cases of bromide toxicity, this known interaction can be used to increase the excretion of bromide by the administration of high amounts of sodium or ammonium chloride (43), as previously described. Furosemide was also formerly used to promote bromide diuresis in cases of bromism and bromoderma (49, 50). Its exact impact on bromide clearance is still unclear (76).

Since bromide is eliminated by the kidneys, renal disease can lead to an inappropriate rise in bromide serum concentration and consequent bromide toxicity (50). Regular monitoring of the kidney function is therefore recommended in patients receiving bromide treatment (50).

## 9 Discussion

Managing epilepsy effectively in veterinary patients remains a challenging aspect of veterinary neurology since poorly controlled seizures can severely impact both the patient’s and the owner’s quality of life. Additionally, a 20–30% of epileptic dogs are refractory to treatment (23, 27, 93, 94), a phenomenon also observed in human medicine (95).

It’s no surprise that the field of epilepsy continues to receive extensive research efforts in order to find additional therapeutic options that can potentially enhance the management of this condition.

Since its introduction by Schwartz-Porsche and Jürgens (26) that bromide continued to have an important role in the treatment of epileptic dogs, refractory to phenobarbital therapy.

Bromide’s long half-life can be seen as an advantage since the administration of loading doses can still allow the clinician to provide the patient with therapeutic serum concentrations in a timely manner (14) and maintenance doses can be performed only once daily, possibly contributing to owner’s compliance.

Bromide follows phenobarbital and imepitoin (for idiopathic epilepsy) as the ASM with “moderate recommendation and most likely to be effective treatment” when used in monotherapy (11).

Use in monotherapy can be particularly interesting in patients that have a contraindication for the treatment with first line ASM (e.g., phenobarbital in case of hepatic disease; structural epilepsy in case of imepitoin). Despite this, it is important to note that evidence to support the use of bromide in monotherapy is limited to one single study by Boothe et al. (76).

Similarly, the currently accepted therapeutic ranges as an “add-on” treatment or in monotherapy are only based on a few studies where these serum concentrations appeared to be effective (16, 23, 26, 27). Diets were also not uniform throughout the patients of these studies, possibly impacting bromide half-life and consequently the level of maintenance dose required.

Future prospective studies assessing bromide efficacy as monotherapy in epileptic dogs receiving a stable and uniform diet could help to better clarify its possible role as a possible first-line ASM and deepen our understanding of recommended serum therapeutic ranges.

Nonetheless, the noticeable variable sensitivity to bromide between different canine patients (40, 63), highlights the need for a tailored and individualized treatment plan, with the currently known therapeutic ranges and suggested doses to be used as an initial guide.

Due to the challenges presented in assessing serum bromide concentrations, clinicians should aim to work with a trusted laboratory and avoid comparing results obtained from different laboratories. Furthermore, as the Tri-gold measurement method carries a risk in getting incorrect values, it is advised to use laboratories that use mass spectrometry as a measurement method (96).

Owners and clinicians alike should be aware of the impact abrupt changes in chloride intake can have in seizure control. This is important to remember when these patients receive treatment for other conditions, particularly when intravenous fluid therapy is used. Bromide serum concentration can drop in a matter of hours possibly leading to breakthrough seizures (68). Bromide concentration should be closely monitored, and dose adjustment might be required during these periods.

Adverse effects seem to be the main disadvantage associated with bromide treatment, but these appear to be better controlled and tolerable in dogs when compared to people.

Although some authors advise that gastric irritation caused by bromide and consequent vomiting can be limited or controlled by dividing daily dosage or with its administration in association with food (28, 44, 63), to the authors’ knowledge, prospective studies assessing the effect of these measures on the frequency of gastrointestinal side effects are still lacking.

In human patients, neither phenobarbital nor bromide were associated with the development of pancreatitis (97). Studies assessing the risk of pancreatitis in bromide treated dogs are limited (65–67) and although this still appears to be a possible complication of the use of this medication, evidence for a causal effect has not yet been obtained. It is currently unclear whether the possible risk of pancreatitis might be due to onset of pica and polyphagia, leading to alimentary indiscretion and associated complications (63). In any case, it seems wise to monitor for signs of pancreatitis in canine patients treated with bromide. The value of monitoring the patient’s triglyceridemia in preventing or anticipating signs of pancreatitis is also unknown and could reveal an interesting field for future studies.

Due to the known possible weight changes, body weight should also be closely monitored.

In feline patients, use of bromide cannot be advised due to the severity of adverse effects. Studies assessing bromide efficacy are also (and should continue to be) limited due to this (41, 62).

To the authors knowledge, studies assessing bromide use and efficacy in horses are not available and the possible role of bromide as a treatment of epileptic seizures in this species is still unknown.

Treating epilepsy requires tailor-made medicine. Bromide remains a cornerstone in the treatment of canine epilepsy since its introduction in the 1990s. Despite its widespread use, studies supporting the currently recommended serum therapeutic concentrations and its efficacy when used as monotherapy are still scarce. Future prospective research on bromide use, monitoring, interactions and side effects could enhance our understanding of this medication even further and ultimately help empowering clinicians and owners with greater confidence and efficacy in the management of canine epilepsy. Nonetheless, we can identify some potential good, bad and ugly aspects of the use of this medication. The good is that bromide has proved efficacy as an add-on treatment in cases of canine refractory idiopathic epilepsy and might also serve as an alternative first-line ASM in selected cases. The currently known challenges in measuring and monitoring bromide serum concentrations, make it difficult to tailor dosages to the individual patient and represent one of the downsides (or bad aspects) of its use. Finally, the ugly side of bromide use seems mainly associated with cases where clinicians failed to adequately adjust the treatment to the patients’ needs leading to a wide range of adverse effects. These might impact the quality of life of both dogs and owners to an unacceptable degree. The severity of adverse effects can be such that bromide was largely abandoned in human medicine and is currently contraindicated in cats. Continuous and accurate monitoring of bromide serum concentrations is necessary to maximize its therapeutic properties and ensure its safe use.

## Author contributions

DG: Conceptualization, Writing – original draft, Writing – review & editing. PM: Conceptualization, Supervision, Writing – review & editing. GC: Conceptualization, Supervision, Writing – review & editing.
